# Clinical Significance of Variable Histomorphologic Findings Related to Mucosal Inflammation in Negative Appendectomy

**DOI:** 10.3390/jcm10174030

**Published:** 2021-09-06

**Authors:** Euno Choi, Youngeun Yoo, Ji Min Kim, Sun Hee Sung, Dakeun Lee, Sanghui Park

**Affiliations:** 1 Department of Pathology, Ewha Womans University College of Medicine, Seoul 07985, Korea; euno1103@hanmail.net (E.C.); k-jm0309@hanmail.net (J.M.K.); sunhsung@ewha.ac.kr (S.H.S.); 2 Department of Pathology, Asan Medical Center, University of Ulsan College of Medicine, Seoul 05505, Korea; yeyoo88@gmail.com; 3 Department of Pathology, Ajou University School of Medicine, Suwon 16499, Korea; dakeun@gmail.com

**Keywords:** negative appendectomy, appendicitis inflammatory response (AIR) score, mucosal inflammation, high neutrophil score, surface epithelial flattening

## Abstract

The aim of the study was to investigate the clinical significance of various histomorphologic findings related to mucosal inflammation in negative appendectomy. We reviewed histopathologic findings of 118 negative appendectomies and correlated them with the appendicitis inflammatory response (AIR) score and appendiceal diameter. Among 118 patients with negative appendectomy, 94 (80%), 73 (78%) and 89 (75%) patients displayed mucosal inflammation, high neutrophil score (neutrophil count ≥10/5 high power field and surface epithelial flattening, respectively. Out of 118 patients with negative appendectomy, mucosal inflammation, high neutrophil score and surface epithelial flattening were associated with higher risk group according to the appendicitis inflammatory response (AIR) score (*p* < 0.05, respectively). In addition, mucosal inflammation, high neutrophil score and surface epithelial flattening were frequently detected in 118 negative appendectomies, compared with 24 incidental appendectomies (*p* < 0.05, respectively). In an analysis of 77 negative appendectomy patients with appendiceal diameter data available, increased appendiceal diameter was positively correlated with luminal inflammation, high neutrophil score and surface epithelial flattening (*p* < 0.05, respectively). In conclusion, mucosal inflammation, high neutrophil score and surface epithelial flattening in negative appendectomy may be relevant to patients’ signs and symptoms, especially in cases with no other cause of the abdominal pain.

## 1. Introduction

The diagnosis of acute appendicitis (AA) is often challenging due to ambiguous symptoms that can result from other diseases. Several clinical scoring systems have been proposed to improve the diagnostic accuracy of AA [1,2,3], and the Alvarado score is most well established. The Alvarado score predicts likelihood of AA, based on three symptoms (migratory pain, anorexia and nausea or vomiting), three signs (right lower quadrant pain, rebound tenderness and elevated temperature) and two laboratory findings (leukocytosis and shift to left). Patients with an Alvarado score higher than seven have a 93% probability of being diagnosed with AA [1]. However, the appendicitis inflammatory response (AIR) score, which incorporates C-reactive protein (CRP) and gradation of RLQ pain, was proven to outperform the Alvarado score in several studies [3,4,5]. Particularly, in the previous study on the diagnostic performance of the risk stratification using AIR score, intermediate and high risk (AIR score of 5 or more) showed high sensitivity for AA and high risk (AIR score of 9 or more) was very specific for AA [6].

The accurate diagnosis of AA in patients presenting with atypical clinical symptoms is challenging, sometimes resulting in negative appendectomy, which is defined as the surgical removal of a histologically normal appendix [7]. Historically, a negative appendectomy rate of 15~25% has been found acceptable to minimize the risk of complications from delayed or missed diagnosis of AA, such as perforation, abscess formation and peritonitis [8]. The use of imaging modalities, such as ultrasonography (US) or computed tomography (CT), have led to a decreased negative appendectomy rate, especially among women [9,10,11].

As a rule, in the pathologic perspective, the presence of transmural neutrophilic infiltration is a required finding for the diagnosis of AA [7,12,13]. Occasionally, in surgical specimens resected with clinical suspicion of AA, neutrophilic infiltration is confined to the mucosa with and without mucosal erosion, which are considered as pathologic features that may be seen in early appendicitis [14,15]. However, a controversy in regard to this entity still remains, because the association between these histologic features and patients’ symptoms is vague, and these histologic features are frequently detected in surgical specimens that are incidentally removed during non-appendiceal surgery [7,16]. Thus, these histologic features are recognized as nonspecific findings related to normal variant by several authors [16,17]. For this reason, the definition of negative appendectomy can be different among various surgeons and pathologists, when neutrophilic infiltration is limited to the mucosa in the surgically removed appendix [7]. In addition to mucosal inflammation, surface epithelial flattening, mural eosinophilic infiltration, lymphoid follicular hyperplasia and epithelial hyperplastic change are morphologic changes seen in negative appendectomy specimens, of which the clinical significance is also unclear [7].

In the current study, we defined surgically resected appendix with a clinical suspicion of AA, but lacking transmural neutrophilic infiltration as negative appendectomy [7,13]. Additionally, we aimed to retrospectively evaluate correlations of variable histomorphologic changes with AIR score and preoperative imaging findings in negative appendectomy specimens to identify the clinical significance of the morphologic alterations observed in negative appendectomy.

## 2. Materials and Methods

### 2.1. Case Selection

Retrospective chart review was conducted for 2804 consecutive cases of surgically resected appendices between 2007 and 2012 with a clinical suspicion of AA. A total of 2804 patients presented with sudden-onset abdominal pain, specifically right lower quadrant pain.

A diagnosis of AA was confirmed when transmural infiltration of neutrophils was seen in resected appendices. Hematoxylin and eosin (H&E) stained slides of 2804 appendectomy cases were reviewed in a blinded fashion. Of 2804 cases, 118 negative appendectomy cases that failed to show transmural neutrophilic infiltration were selected for this study. Twenty-four cases of incidental appendectomy were used as negative controls. Clinical data regarding sex, age, radiologic findings and AIR scores were extracted from medical records. Patients were classified into two groups based on AIR score as follows: low risk group (score < 5), intermediate risk group (5 ≤ score < 8). None of the patients belonged to the high risk group (score ≥ 8). This study was approved by the Institutional Review Board of Ewha Womans University Mokdong Hospital.

### 2.2. Histologic Evaluation

**Figure 1:**
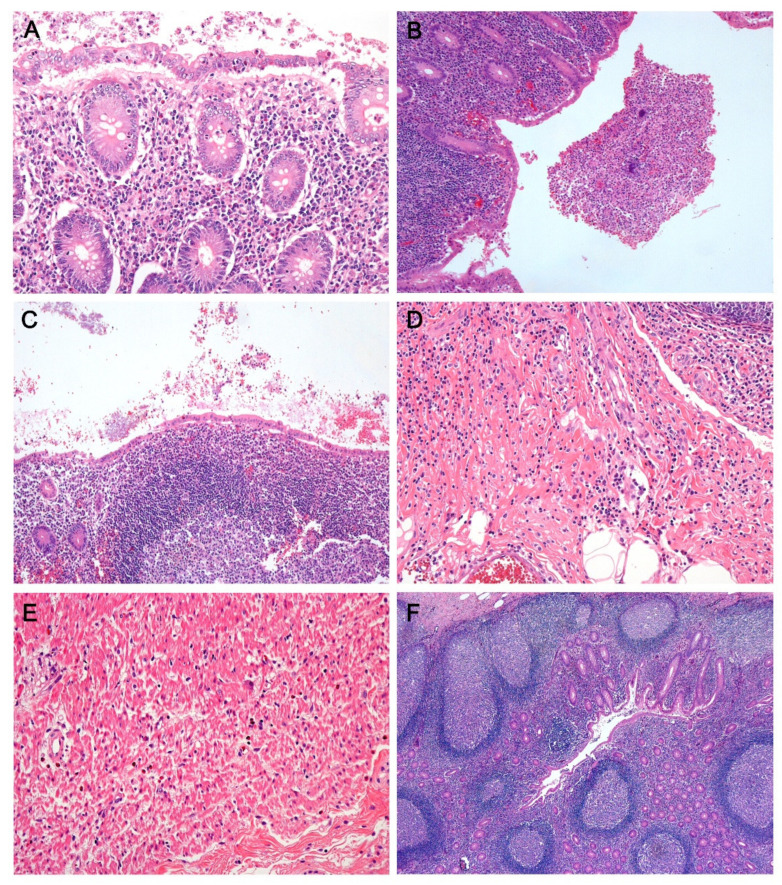
Variable histopathologic findings in negative appendectomy specimens (hematoxylin and eosin. (**A**) Mucosal inflammation. Neutrophilic infiltration is observed within the crypt epithelium and lamina propria. (**B**) Luminal inflammation. Intraluminal neutrophilic aggregates are seen. (**C**) Mucosal flattening. The surface mucosa is lined by cuboidal or flattened epithelial cells with loss of goblet cells. (**D**) Submucosal inflammation. Mixed inflammatory cells, including neutrophils, infiltrate the submucosal layer without involvement of the proper muscle layer. (**E**) Eosinophilic infiltration in the proper muscle layer. (**F**) Lymphoid follicular hyperplasia. (**A**,**D**,**E**: ×200, **B**,**C**: ×100, **F**: ×40).

In the series of 118 negative appendectomies, histologic findings were assessed by two pathologists (E.C. and S.P.) on each case using the following parameters: mucosal inflammation (neutrophilic infiltration in lamina propria accompanied by cryptitis/crypt abscess), luminal inflammation (intraluminal neutrophilic discharge), submucosal inflammation (neutrophilic infiltration in submucosa), mucosal erosion, surface epithelial flattening and epithelial hyperplastic change [7]. In addition, for cases harboring mucosal inflammation, the number of neutrophils was measured in the mucosa; cases were classified into the high neutrophil score when more than 10 neutrophils/5 high power field (HPF) were found in the mucosa [18]. The number of eosinophils infiltrating the muscularis propria was measured and more than 10 eosinophils/mm^2^ (4 HPF) was defined as the high eosinophil score [7]. Lymphoid follicular hyperplasia was defined as hyperplastic lymphoid follicles harboring more than 10 lymphoid nodules, in which each follicle had a diameter 2 mm or larger (Figure 1) [19].

### 2.3. Radiologic Findings

The records for radiologic findings of 77 patients having data on appendiceal diameters were reviewed. Appendiceal diameters were subdivided into three categories, as follows: appendiceal diameter <6 mm, normal; 6 ≤ appendiceal diameter < 7 mm, borderline dilatation; appendiceal diameter ≥7 mm, dilatation compatible with a diagnosis of AA [20].

### 2.4. Statistical Analysis

Data analysis was performed using SPSS statistical software (version 21.0; IBM SPSS Inc., Armonk, NY, USA). Pearson χ^2^ test or Spearman correlation analysis was used to compare categorical variables. A value of *p* < 0.05 was considered statistically significant. 

## 3. Results

### 3.1. Patient Baseline Characteristics

The 118 patients with negative appendectomy included 65 males (55%) and 53 females (45%) with a median age of 19 years (range: 4–71 years). Of these 118 patients, 61 (52%) were younger than 19 years. The AIR scores of 118 patients ranged from 0 to 7 with an overall mean value of 2.8. Ninety-nine patients (84%) were classified as belonging to the low risk group and 19 patients (16%) to the intermediate risk group based on the risk stratification using AIR score. Mesenteric lymphadenopathy was detected in 16 patients (14%), most of whom were pediatric patients (age: 3–18 years). Twenty-four patients who underwent incidental appendectomy were composed of 17 males and 7 females with a median age of 13 years (range: 0–65 years). Most patients (92%, 22/24) were in the low risk group and the remaining two patients belonged to the intermediate risk group. In these 24 patients, intussusception (33%, 8/24) was the most frequent cause of the incidental appendectomy, followed by non-appendiceal tumor surgery (25%, 6/24), surgical repair of intestinal obstruction or midgut volvulus (29%, 7/24), gynecologic surgery for pelvic inflammatory disease (8%, 2/24), and abdominal stab wound (4%, 1/24).

Preoperative CT and US were performed in 75 patients (64%) and 41 patients (35%), respectively. One patient underwent both CT and US imaging and another patient had an appendectomy without any preoperative imaging under suspicion of AA during cesarean section. In the 77 patients with preoperative CT or US imaging, and for whom appendiceal diameters data were available, the diameter of the appendix was <6 mm (normal appendix) in 13 patients (17%), 6–6.9 mm (borderline dilatation) in 27 patients (35%) and 7–9 mm (dilatation compatible with a diagnosis of AA) in 37 patients (48%).

### 3.2. Correlation between Histomorphologic Findings and AIR Score-Based Risk Stratification in Negative Appendectomy

**Figure 2:**
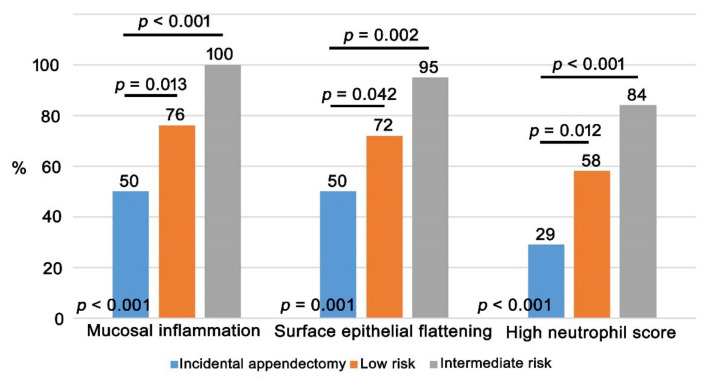
Comparison of histologic findings between incidental appendectomy cases and negative appendectomy cases that were classified according to AIR score-based risk stratification. Negative appendectomy cases with low risk and intermediated risk displayed more frequent mucosal inflammation, surface epithelial flattening and high neutrophil score than incidental appendectomy cases (*p* < 0.05, respectively). Additionally, positive linear correlations were shown in the comparison of these histologic features among incidental appendectomy cases, negative appendectomy cases with low risk and intermediate risk (*p* < 0.05, respectively), of which *p* value is presented in the bottom of each graph.

Among the 118 patients with negative appendectomies, 94 (80%) showed mucosal inflammation. Of the 94 patients with mucosal inflammation, luminal inflammation and submucosal inflammation were found in 18 and 28 patients, respectively. Twenty-four patients (20%) lacking mucosal inflammation had neither luminal inflammation nor submucosal inflammation. Out of the 118 patients with negative appendectomies, mucosal erosion and surface epithelial flattening were detected in 29 (25%) and 89 (75%) patients, respectively. When classifying the 118 patients into two groups based on the presence or absence of mucosal inflammation, the group having mucosal inflammation displayed more frequent luminal inflammation, submucosal inflammation, mucosal erosion, surface epithelial flattening, high neutrophil score and high eosinophil score (*p* < 0.05, each) (Table 1). Lymphoid follicular hyperplasia was observed in 37 patients (31%) and was more commonly observed in pediatric patients than in adult patients (*p* < 0.05). Other histologic parameters including mucosal inflammation, submucosal inflammation, luminal inflammation, mucosal erosion, surface epithelial flattening and epithelial hyperplastic change were not significantly different between pediatric (3–18 years) and adult patients. In addition, mucosal inflammation, surface epithelial flattening and high neutrophil score were more common in 118 patients with negative appendectomies compared with the 24 who underwent incidental appendectomy (*p* < 0.05) (Table 1). When comparing incidental appendectomy cases and negative appendectomy cases classified by AIR score-based risk stratification, negative appendectomy cases with low risk and intermediated risk exhibited more frequent mucosal inflammation, surface epithelial flattening and high neutrophil score compared with incidental appendectomy cases (*p* < 0.05, respectively). Regarding the presence of these histologic features, an increasing linear trend was shown among incidental appendectomy patients and negative appendectomy patients with low risk and intermediate risk (*p* < 0.05) (Figure 2).

### 3.3. Correlation between Histomorpholgic Findngs and Appendiceal Diameter in Negative Appendcetomy

In an analysis of the 77 patients with available appendiceal diameter data on preoperative radiologic reports, increased appendiceal diameter was positively correlated with luminal inflammation, high neutrophil score, surface epithelial flattening and lymphoid follicular hyperplasia (*p* < 0.05) (Table 2). No patients in the intermediate risk group according to AIR score had an appendix diameter <6 mm. In particular, the 45 pediatric patients (age: 3–18 years) exhibited more dilated appendices compared with 32 adult patients (*p* < 0.05) (Table 2).

## 4. Discussion

Removing a pathologically normal appendix is a common surgical issue in AA and is defined as negative appendectomy [7]. Negative appendectomy remains a concern for surgeons due to the associated risks of unnecessary anesthesia and surgical complications [21,22]. Furthermore, in a significant number of patients who underwent a negative appendectomy, no other cause of the abdominal pain could be found clinically [7]. For this reason, substantial attention has been drawn to histopathologic changes in negative appendectomy specimens that could explain abdominal pain.

Historically, there has been long-standing controversy as to whether neutrophilic infiltration confined to the mucosa should be considered early appendicitis or nonspecific histologic findings in negative appendectomy specimens. So far, many pathologists agree that the diagnosis of acute appendicitis requires intramural neutrophilic infiltration, because mucosal neutrophilic infiltration can often be shown in incidental appendectomy specimens [7,16]. In the current study, we correlated the AIR score and variable histologic findings in negative appendectomy specimens according to the presence or absence of mucosal inflammation and compared the histologic findings between negative appendectomy and incidental appendectomy to identify the clinical significance of various histologic features in negative appendectomy specimens.

This study showed that the presence of mucosal inflammation was associated with higher risk according to AIR score and histologic severity in patients with negative appendectomy. In our study, negative appendectomy patients having mucosal inflammation were at higher risk according to AIR score than those lacking mucosal inflammation. This suggests that more severe patient symptoms can be attributed to a higher degree of mucosal inflammation in negative appendectomy. Our results are in agreement with a previous report by Mizumoto et al. [23], which suggested that patients with mucosal appendicitis had higher mean Alvarado scores compared with those who had a histologically normal appendix. Additionally, various histomorphologic changes, including luminal inflammation, submucosal inflammation, high neutrophil score, high eosinophil score, erosion and surface epithelial flattening, were more frequently observed in negative appendectomy patients with mucosal inflammation than in those without mucosal inflammation. Indeed, the clinicopathologic significance of these histologic features is unclear. However, in a previous study, luminal inflammation with local mucosal erosion can be found in patients presenting with right lower quadrant pain [7]. Intramural eosinophils infiltration is considered an early event of AA, which reflects a type I hypersensitivity to an allergen [24,25]. In addition, in a previous study, surface epithelial flattening was proven to be a feature which is detected in otherwise normal appendices with clinical features of AA more often than in incidental appendectomy specimens [26]. Thus, when no other cause of abdominal pain is clearly identified, mucosal inflammation of the appendix can be considered a distinct pathologic entity, in spite of the absence of transmural neutrophilic infiltration.

There has been controversy regarding the association between various histologic changes in negative appendectomy and patients’ symptoms because these findings can often be seen in incidental appendectomy [7,16,23]. Several histologic features, including mucosal inflammation, intraluminal inflammation and focal mucosal erosion, were known to be commonly shown in incidental appendectomy specimens. On that basis, these histologic findings were not considered pathologic findings related to the symptoms of patients who underwent appendectomy with a clinical suspicion of AA. In the current study, mucosal inflammation, surface epithelial flattening, and high neutrophil score were more frequently observed in negative appendectomy specimens than in incidental appendectomy specimens. Additionally, the presence of these histologic findings showed strong positive linear correlations among incidental appendectomy patients and negative appendectomy patients with AIR score-based low risk and intermediate risk. Furthermore, most patients who underwent negative appendectomy experienced symptom relief after surgery and did not revisit the hospital with the same symptoms. Therefore, these findings indicate that the clinical symptoms of patients who undergo negative appendectomy may be ascribed to several histologic changes, including mucosal inflammation, surface epithelial flattening and high neutrophil score, when no other cause of abdominal pain is found. To the best of our knowledge, this is the first study to demonstrate an association between histologic findings and a clinical scoring system for appendicitis with a comparison of histologic features between negative appendectomy and incidental appendectomy.

With regard to radiologic findings, the results of the current study demonstrated that an increased appendiceal diameter on preoperative image was related to mucosal inflammatory changes or mucosal flattening in negative appendectomy specimens. All patients who underwent negative appendectomy with an appendiceal diameter of less than 6 mm on preoperative images belonged to the low risk group according to AIR score. These findings indicated that acute mucosal inflammatory or architectural changes may contribute to symptoms of appendicitis and altered appendiceal diameter on imaging. Several studies have shown that preoperative imaging can reduce negative appendectomy rates [9,10,27]. However, the association between preoperative image findings and histologic changes of the appendix in negative appendectomy specimens has not been explored. Further studies are warranted to investigate the link between appendiceal diameter and histologic severity in negative appendectomy specimens.

Our study has certain limitations. First, in a total of 118 negative appendectomy cases, the entire appendix was not submitted to the search for the focal presence of transmural neutrophilic infiltration. Second, the same criteria were used for evaluating appendiceal diameter whether patients underwent CT or US. Orscheln et al. [28] demonstrated that appendiceal diameter differs between CT and US by about 1mm in cases not diagnosed with acute appendicitis. In our series, 75 patients (64%) underwent CT, 41 (35%) underwent US, and 1 (1%) underwent both. Considering the small sample size, we did not differentiate between CT and US in the evaluation of the appendiceal diameters, which may have resulted in measurement bias.

## 5. Conclusions

Mucosal inflammation, high neutrophil score and surface epithelial flattening can be considered pathologic phenomena that may contribute to clinical signs and symptoms in patients who undergo negative appendectomy. In addition, luminal inflammation, high neutrophil score and surface epithelial flattening can be reflected in increased appendiceal diameter on preoperative imaging. Thus, histomorphologic changes, including mucosal inflammation, high neutrophil score and surface epithelial flattening in negative appendectomy specimens, may contribute to patients’ signs and symptoms, especially in cases in which no other cause of the abdominal pain can be identified.

## Figures and Tables

**Table 1:** Comparison of clinicopathologic features between negative appendectomy and incidental appendectomy cases.

		NA			
Variables, *n* (%)	IA	NA without MI	NA with MI	*p* Value	
	(*n* = 24)	(*n* = 24)	(*n* = 94)	a	b
Age, years (median, range)	13 (0–65)	20 (4–71)	18.5 (4–67)		
Sex				0.155	0.72
Male	17 (71%)	14 (58%)	51 (54%)		
Female	7 (29%)	10 (42%)	43 (46%)		
Risk according to AIR score				0.328	0.016 *
Low	22 (92%)	24 (100%)	75 (80%)		
Intermediate	2 (8%)	0	19 (20%)		
Mucosal inflammation				0.002 *	<0.001 *
Absent	12 (50%)	24 (100%)	0		
Present	12 (50%)	0	94 (100%)		
Luminal inflammation				0.729	0.02 *
Absent	21 (87%)	24 (100%)	76 (81%)		
Present	3 (13%)	0	18 (19%)		
Submucosal inflammation				0.225	0.002*
Absent	21 (87%)	24 (100%)	66 (70%)		
Present	3 (13%)	0	28 (30%)		
Mucosal neutrophil count				0.003 *	<0.001 *
Low (<10/5 HPF)	17 (71%)	24 (100%)	21 (22%)		
High (≥10/5 HPF)	7 (29%)	0	73 (78%)		
Intramuscular eosinophil count				0.172	0.031 *
Low (≤10/4 HPF)	21 (87%)	22 (92%)	66 (70%)		
High (>10/4 HPF)	3 (13%)	2 (8%)	28 (30%)		
Mucosal erosion				0.197	0.009 *
Absent	21 (87%)	23 (96%)	66 (70%)		
Present	3 (13%)	1 (4%)	28 (30%)		
Surface epithelial flattening				0.012 *	<0.001 *
Absent	12 (50%)	15 (62%)	14 (12%)		
Present	12 (50%)	9 (38%)	80 (68%)		
LFH				0.303	0.213
Absent	19 (79%)	19 (79%)	62 (66%)		
Present	5 (21%)	5 (21%)	32 (34%)		
Hyperplastic epithelial change				0.878	0.852
Absent	22 (92%)	22 (92%)	85 (90%)		
Present	2 (8%)	2 (8%)	9 (10%)		

Abbreviations: NA, negative appendectomy; MI, mucosal inflammation; AIR, acute inflammatory response; LFH, lymphoid follicular hyperplasia; HPF, high power field. a: *p* value for the comparison between patients with incidental appendectomy and patients with negative appendectomy b: *p* value for the comparison between negative appendectomy patients with mucosal inflammation and those without mucosal inflammation * *p* value < 0.05.

**Table 2:** Correlation between appendiceal diameter and AIR score and histologic parameters in 77 patients having available data for appendiceal diameters.

Variables, *n* (%)	Appendiceal Diameter			*p* Value
	<6 mm	6–6.9 mm	7–9 mm	
	(*n* = 13)	(*n* = 27)	(*n* = 37)	
**Risk according to AIR score**				0.144
Low risk (1–4)	13 (100%)	23 (85%)	30 (81%)	
Intermediate risk (5–7)	0	4 (15%)	7 (19%)	
Age				0.185
Pediatric (3–18 years)	5 (38%)	18 (67%)	24 (65%)	
Adult	8 (62%)	9 (33%)	13 (35%)	
Histologic parameters				
Mucosal inflammation				0.116
Absent	4 (31%)	8 (30%)	4 (11%)	
Present	9 (69%)	19 (70%)	33 (89%)	
Luminal inflammation				0.029 *
Absent	13 (100%)	24 (89%)	26 (70%)	
Present	0	3 (11%)	11 (30%)	
Submucosal inflammation				0.105
Absent	8 (62%)	24 (89%)	26 (70%)	
Present	5 (38%)	3 (11%)	11 (30%)	
Mucosal neutrophil count				0.034 *
Low (<10/5 HPF)	6 (46%)	15 (56%)	9 (24%)	
High (≥10/5 HPF)	7 (54%)	12 (44%)	28 (76%)	
Intramuscular eosinophil count				0.21
Low (≤10/4 HPF)	12 (92%)	18 (67%)	26 (70%)	
High (>10/4 HPF)	1 (8%)	9 (33%)	11 (30%)	
Mucosal erosion				0.408
Absent	11 (85%)	19 (70%)	24 (65%)	
Present	2 (15%)	8 (30%)	13 (35%)	
Surface epithelial flattening				0.004 *
Absent	8 (62%)	8 (30%)	5 (14%)	
Present	5 (38%)	19 (70%)	32 (86%)	
LFH				0.028 *
Absent	10 (77%)	22 (81%)	19 (51%)	
Present	3 (23%)	5 (19%)	18 (49%)	
Hyperplastic epithelial change				0.995
Absent	12 (92%)	25 (93%)	34 (92%)	
Present	1 (8%)	2 (7%)	3 (8%)	

Abbreviations: AIR, acute inflammatory response; LFH, lymphoid follicular hyperplasia; HPF, high power field. * *p* value < 0.05.

## Data Availability

Access to the data presented in this study can be obtained from the corresponding author upon reasonable request.
